# Overview of Oxidative Stress Response Genes in Selected Halophilic Fungi

**DOI:** 10.3390/genes9030143

**Published:** 2018-03-06

**Authors:** Cene Gostinčar, Nina Gunde-Cimerman

**Affiliations:** Department of Biology, Biotechnical Faculty, University of Ljubljana, Jamnikarjeva 101, SI-1000 Ljubljana, Slovenia; nina.gunde-cimerman@bf.uni-lj.si

**Keywords:** oxidative stress, reactive oxidative species, halophilic fungi, halotolerant fungi, peroxidase, catalase, *Wallemia ichthyophaga*, *Hortaea werneckii*, *Aureobasidium pullulans*

## Abstract

Exposure of microorganisms to stress, including to high concentrations of salt, can lead to increased production of reactive oxygen species in the cell. To limit the resulting damage, cells have evolved a variety of antioxidant defenses. The role of these defenses in halotolerance has been proposed before. Whole genome sequencing for some of the most halotolerant and halophilic fungal species has enabled us to investigate the possible links between oxidative and salt stress tolerance on the genomic level. We identified genes involved in oxidative stress response in the halophilic basidiomycete *Wallemia ichthyophaga*, and halotolerant ascomycetous black yeasts *Hortaea werneckii* and *Aureobasidium pullulans*, and compared them to genes from 16 other fungi, both asco- and basidiomycetes. According to our results, *W. ichthyophaga* can survive salinities detrimental to most other organisms with only a moderate number of oxidative stress response genes. In other investigated species, however, the maximum tolerated salinity correlated with the number of genes encoding three major enzymes of the cellular oxidative stress response: superoxide dismutases, catalases, and peroxiredoxins. This observation supports the hypothetical link between the antioxidant capacity of cells and their halotolerance.

## 1. Introduction

Production of reactive oxygen species (ROS) is one of the fundamental characteristics of oxygen metabolism across the tree of life. As a consequence, cells have evolved mechanisms, which in normal circumstances efficiently limit the damage these chemicals can cause. However, the balance between the production of ROS and the antioxidant defenses can be disturbed, leading to oxidative stress. Such imbalances can be triggered by various types of environmental stress, among them heat and dehydration [1,2]. High environmental salinity has been shown to trigger ROS accumulation in various eukaryotes [3,4]. Unsurprisingly, the responses to both osmotic and oxidative stress are overlapping [5].

Three major ROS are encountered in the cell [6]. Reduction of molecular oxygen (O_2_) produces a superoxide anion (O_2_^−^), which is converted by superoxide dismutases to hydrogen peroxide (H_2_O_2_) and this is fully reduced by catalases or glutathione peroxidases to water, or partially reduced (via Fenton reaction catalyzed by ferrous ions) to hydroxyl radical (HO**^•^**). The damage caused by O_2_^−^ is primarily targeted at proteins with prosthetic Fe-S groups. HO**^•^** are extremely oxidizing, causing indiscriminate damage to all major groups of biomolecules. As a consequence, they are also short-lived and the damage they cause is spatially limited. Compared to this, H_2_O_2_ is relatively stable and is a non-charged small molecule, which can diffuse through biological membranes, while in the cell it primarily damages Fe-S proteins [6].

Various ROS in the cell not only differ in their reactivity, longevity and types of damage they cause but also in the detoxification mechanisms that the cells use for their removal [7]. In addition to the above-mentioned superoxide dismutases, catalases and peroxidases, other important enzymes in fungi are peroxiredoxins and the catalase-peroxidases (which both catalyze the reduction of H_2_O_2_), glyoxalases (which detoxify reactive aldehydes), and thioredoxins (which facilitate the reduction of other proteins).

Under certain conditions, non-enzymatic antioxidants are more important than enzymatic, as demonstrated in the case of oxidative stress triggered by ionizing radiation [8]. At least in these conditions the presence of antioxidant enzymes becomes redundant and the major defense system of the cell is triggered by small, non-proteinaceous compounds such as complexes of Mn^2+^, phosphates, certain amino acids and nucleotides [6]. This mechanism is so important that the ratio between manganese bound to proteins (such as Mn-superoxide dismutase) and manganese in small metabolite complexes is a strong predictor of radiation resistance in prokaryotes as well as eukaryotes [9]. Such non-enzymatic mechanisms are difficult, if not impossible to investigate on a genomic level. A very limited insight may perhaps be provided by analysis of genes important for manganese homeostasis, such as the proton-powered manganese importers from the natural resistance-associated macrophage protein (NRAMP) family, which are conserved across all domains of life. In bacterium *Deinococcus radiodurans* this transporter is essential and is thought to be crucial for response to radiation induced oxidative stress [10]. Its expression is upregulated following radiation induced damage [11].

Fungi are good models for investigating the possible link between antioxidant mechanisms and halotolerance. Some fungal species are among the most salt-tolerant organisms known. The black yeast *Hortaea werneckii* can grow almost across the whole salinity gradient: from regular mycological media without added salt, to 28–30% NaCl (w/v) [12]. Basidomycete *Wallemia ichthyophaga*, one of only eight species from a phylogenetically isolated subphylum Wallemiomycotina [13,14] can grow even in media saturated with NaCl [12], and can also grow at concentrations of MgCl_2_ almost twice as large than the most magnesium tolerant bacteria [15]. However, it is unable to grow without salt, a trait that is common in prokaryotic halophiles, but observed in only a few fungal species. In addition to fungi well adapted to high concentrations of salt, some other are less halotolerant, but much better at tolerating other types of stress. A good example of such species is a black yeast *Aureobasidium pullulans*, which has an upper salinity limit at 17% NaCl (w/v), but is also able to tolerate extremely low temperatures, high and low pH, and lack of nutrients, traits that are presumably the basis of its ubiquitous ecology [16].

The link between high salinity and oxidative stress in fungi has not been thoroughly investigated. In *H. werneckii*, oxidative stress was proposed as one of the limiting factors for growth under hypersaline conditions [17]. At 25% NaCl (w/v) the sensitivity of the cells to H_2_O_2_ increased markedly, indicating that in these conditions, ROS defense mechanisms were saturated with intracellular ROS [17]. The expression of genes encoding the aconitase and nicotinamide adenine dinucleotide (NADH) dehydrogenase, two FeS-proteins sensitive to hyperoxia, increased at high salinity [18]. Furthermore, at high concentrations of NaCl an increased expression of genes involved in energy production and oxidative damage protection was observed [19].

Almost two decades of our work with the halotolerant physiology of *H. werneckii* (and for a shorter time also of *A. pullulans* and *W. ichthyophaga*) led us to sequencing their whole genomes [20,21,22]. Here we investigate these genomes for genes involved in the antioxidant response and interpret the findings in the context of other fungal species with varying degrees of salt-tolerance.

## 2. Materials and Methods

Protein sequences of enzymes involved in oxidative stress response were downloaded from the Kyoto Encyclopedia of Genes and Genomes (KEGG) [23] for selected fungal species with sequenced whole genomes representing both ascomycetes and basidiomycetes (Table 1). The following Enzyme Nomenclature (EC number) categories were considered: 1.15.1.1 for superoxide dismutases, 1.11.1.6 for catalases, 1.11.1.15 for peroxiredoxins, 1.11.1.21 for catalase-peroxidases, 2.5.1.18 for glutathione transferases, 4.4.1.5 for glyoxalases I, 3.1.2.6 for glyoxalases II, and 1.11.1.9 for glutathione peroxidases. KEGG orthology (KO) groups K03671 and K12346 were used for thioredoxins and transporters from the NRAMP family, respectively.

These sequences were used as queries for a basic local alignment search tool (BLAST) search against the predicted proteomes of *A. pullulans* (GenBank: AYYB00000000) [20], *H. werneckii* (GenBank: MUNK00000000) [22], and *W. ichthyophaga* (GenBank: APLC00000000) [21] using standalone BLAST+ version 2.7.1 [24] with an E-value threshold of 0.1. A phylogenetic tree was constructed for each enzyme group as described below. *A. pullulans*, *H. werneckii* and *W. ichthyophaga* proteins with unexpected phylogenetic positions or large phylogenetic distances from other proteins in the tree were searched against the non-redundant GenBank protein database [25] and their putative function was either confirmed or they were removed from the dataset on a case-by-case basis. The resulting dataset was again used for the estimation of phylogenies and the selection process was repeated, resulting in a high-confidence protein dataset used in all subsequent analyses.

Phylogenetic analyses were performed on protein sequences aligned with MAFFT v.7.215 [34]. After estimating the best protein evolution model and alpha parameter of the gamma distribution of substitution rate categories with ProtTest v.3.4.2 [35], the phylogenetic trees were generated with the PhyML v.3.3 software [36] with approximate likelihood-ratio test (aLRT) implementation for the calculation of branch supports as Chi-squared-based support.

Correlation analyses were performed in R [37]. Visualization of correlation values as well as of gene homologue numbers were performed with the corrplot package in R [38]. Before analyses the numbers of homologues in *H. werneckii* were divided by two to compensate for its diploid genome, which was formed by hybridization and contains almost all genes in at least two copies [39].

## 3. Results

**Figure 1:**
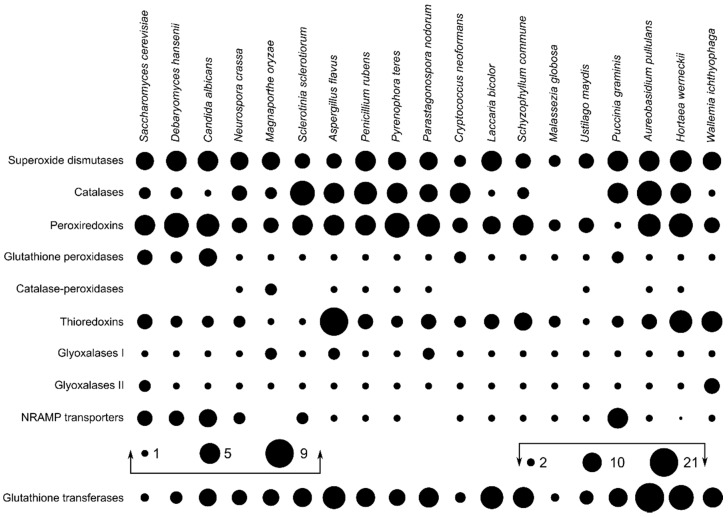
Number of homologues of oxidative stress response genes in selected ascomycetous and basidiomycetous fungi (per haploid genome). The size of dots corresponds to the number of homologues and the absence of a dot marks the absence of a gene in a specific fungus. The numbers of glutathione transferase genes are drawn on a different scale. NRAMP: natural resistance-associated macrophage proteins.

The search for predicted proteins involved in the oxidative stress response resulted in the largest numbers of identified proteins for glutathione transferases (8.66 per species on average), followed by peroxiredoxins, superoxide dismutases, and catalases with (on average) 4.50, 3.97, and 3.32 proteins per haploid genome, respectively (Figure 1). *A. pullulans* and *H. werneckii* had above average numbers of superoxide dismutases, catalases, and peroxiredoxins, but also of catalase-peroxidases, thioredoxins, and glutathione transferases. In contrast, *W. ichthyophaga* contained less than the average number of homologues of most investigated enzymes, with the exception of superoxide dismutases, thioredoxins, and glyoxalases II.

**Figure 2:**
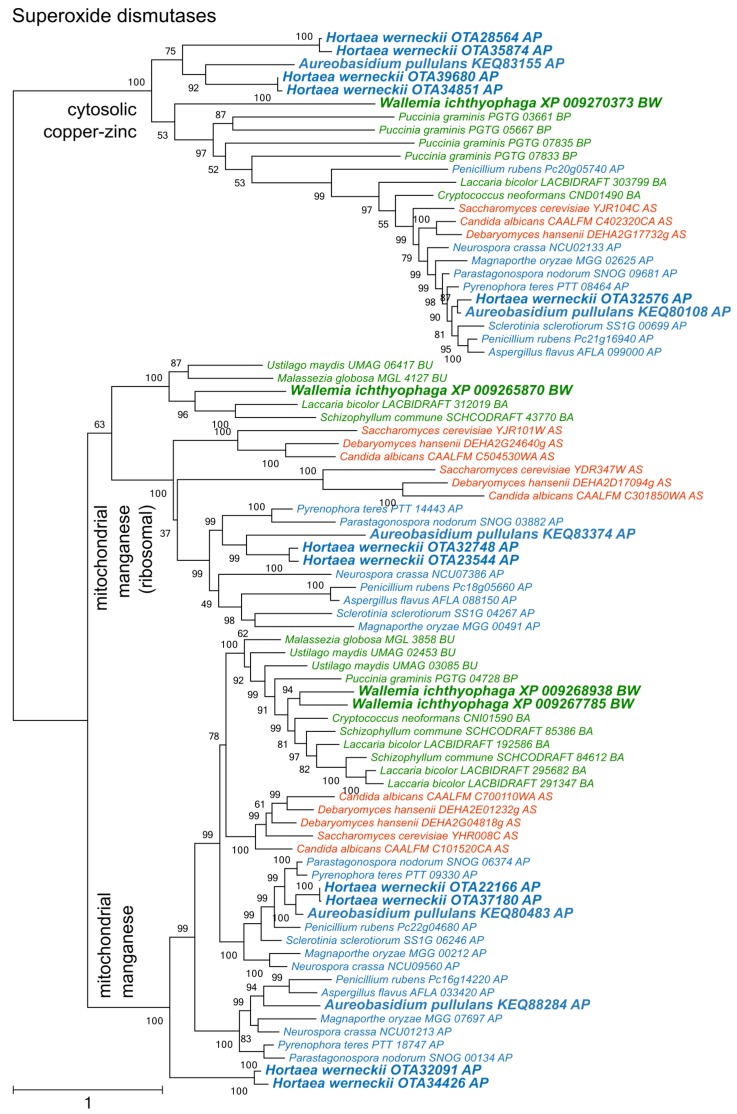
Maximum likelihood phylogeny of genes encoding superoxide dismutases. The phylogeny was estimated by PhyML software with approximate likelihood-ratio test (aLRT) implementation for the calculation of branch supports as Chi-squared based support. Species name is followed by protein accession number and by a two letter description of the larger taxonomic group (BA—Basidiomycota, Agaricomycotina; BU—Basidiomycota, Ustilaginomycotina; BP—Basidiomycota, Pucciniomycotina; BW—Basidiomycota, Wallemiomycotina; AS—Ascomycota, Saccharomycotina; AP—Ascomycota, Pezizomycotina). Green—Basidiomycota, red—Saccharomycotina, blue—Pezizomycotina.

Phylogenetic analysis of superoxide dismutases produced three distinct clusters of proteins (Figure 2), two corresponding to the cytosolic copper/zinc and mitochondrial manganese superoxide dismutases from Saccharomyces cerevisiae, and a third group containing mitochondrial ribosomal proteins with the superoxide dismutase domain. Each group contained representatives from asco- and basidiomycetes, including from *W. ichthyophaga*, *H. werneckii* and *A. pullulans*. Manganese superoxide dismutases from Pezizomycotina formed two separate clusters.

**Figure 3:**
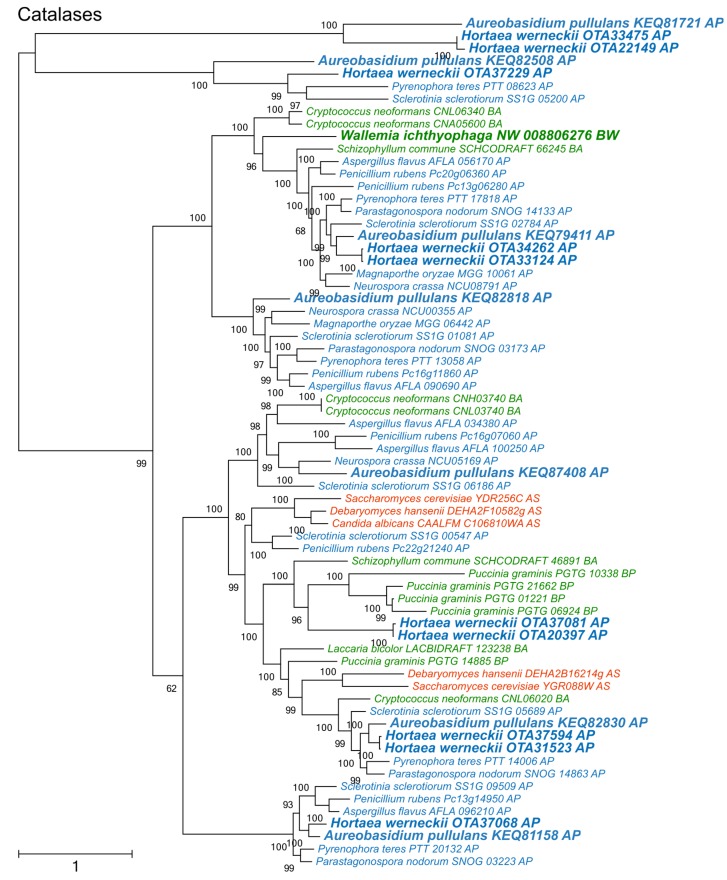
Maximum likelihood phylogeny of genes encoding catalases. The phylogeny was estimated by PhyML software with aLRT implementation for the calculation of branch supports as Chi-squared based support. Species name is followed by protein accession number and by a two letter description of the larger taxonomic group (BA—Basidiomycota, Agaricomycotina; BU—Basidiomycota, Ustilaginomycotina; BP—Basidiomycota, Pucciniomycotina; BW—Basidiomycota, Wallemiomycotina; AS—Ascomycota, Saccharomycotina; AP—Ascomycota, Pezizomycotina). Green—Basidiomycota, red—Saccharomycotina, blue—Pezizomycotina.

Catalases formed two large phylogenetic clusters and a cluster of phylogenetically fairly distant proteins originating from *A. pullulans*, *H. werneckii, Sclerotina sclerotiorum*, and *Pyrenophora teres* (Figure 3). Of the two large clusters, only one contained representatives from Saccharomycotina. The other was dominated by representatives from Pezizomycotina, but also contained proteins from basidiomycetes *W. ichthyophaga, Cryptococcus neoformans*, and *Schizophyllum commune*.

**Figure 4:**
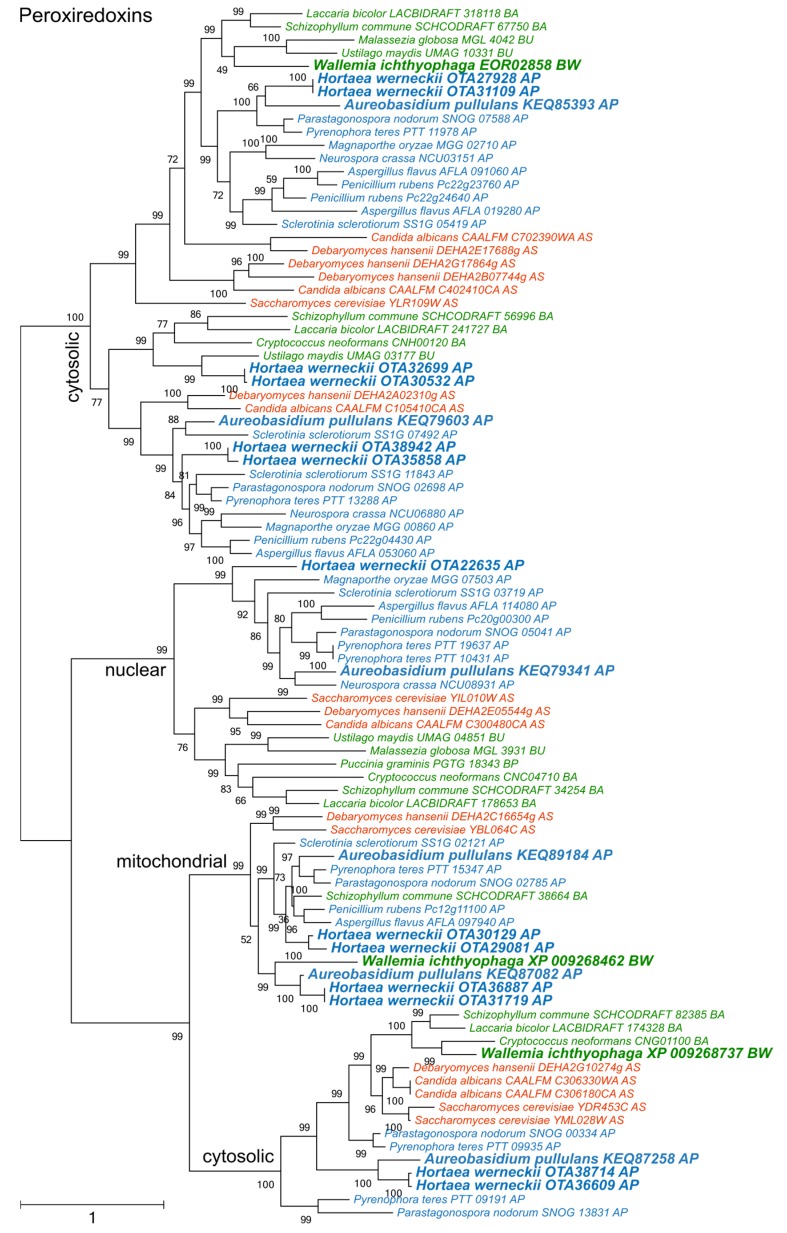
Maximum likelihood phylogeny of genes encoding peroxiredoxins. The phylogeny was estimated by PhyML software with aLRT implementation for the calculation of branch supports as Chi-squared based support. Species name is followed by protein accession number and by a two letter description of the larger taxonomic group (BA—Basidiomycota, Agaricomycotina; BU—Basidiomycota, Ustilaginomycotina; BP—Basidiomycota, Pucciniomycotina; BW—Basidiomycota, Wallemiomycotina; AS—Ascomycota, Saccharomycotina; AP—Ascomycota, Pezizomycotina). Green—Basidiomycota, red—Saccharomycotina, blue—Pezizomycotina.

The diversity of peroxiredoxins surpassed both superoxide dismutases and catalases (Figure 4). Four phylogenetic clusters could be recognized, corresponding to two cytosolic, one mitochondrial, and one nuclear peroxiredoxins. One cytosolic cluster could be further separated into two subclusters, which originated from a duplication that occurred before the separation of asco- and basidiomycetes. *A. pullulans* and *H. werneckii* contained representatives from all clusters, while *W. ichthyophaga* lacked a representative in the group of nuclear enzymes.

**Figure 5:**
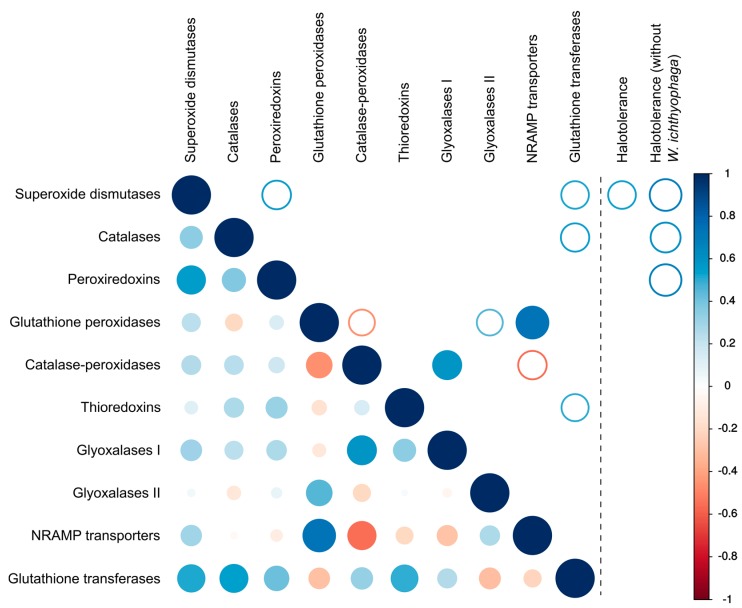
Correlation between the numbers of different genes involved in the oxidative stress response and between the numbers of these genes and halotolerance. Lower-left part of the matrix shows all correlations, upper right part of the matrix shows only the statistically significant correlations (full circles for *p* < 0.01, empty circles for *p* < 0.05). Right panel shows the statistically significant correlations (all with *p* < 0.05) between the number of genes and halotolerance expressed as the maximum salinity growth limit of each investigated species (with and without *Wallemia ichthyophaga* in the dataset).

There was a significant (*p* < 0.01) positive correlation between the number of glutathione peroxidases and transporters of the NRAMP family as well as between catalase-peroxidases and glyoxalases I (Figure 5). Weaker correlations (*p* < 0.05) were observed between the numbers of glutathione peroxidases and glutathione transferases, peroxiredoxins and superoxide dismutases and some other pairs of proteins (Figure 5).

Analysis of the number of genes and the maximum tolerated salt concentration by each species only detected a correlation (*p* < 0.05) between halotolerance and the number of superoxide dismutases. However, after the removal of *W. ichthyophaga* from the dataset halotolerance correlated (*p* < 0.05) with the numbers of three major ROS-detoxification enzymes: superoxide dismutases, catalases and peroxiredoxins. Inclusion or exclusion of *Penicillium rubens* and *Malassezia globosa* (where only halotolerance data for closely related species could be found in the literature) from the dataset did not affect this result.

**Figure 6:**
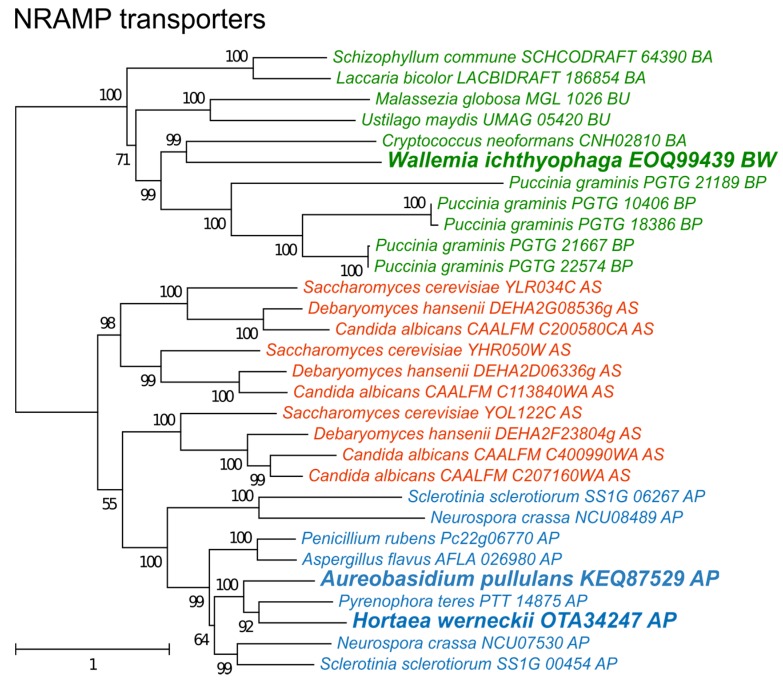
Maximum likelihood phylogeny of genes encoding cation transporters of the NRAMP family. The phylogeny was estimated by PhyML software with aLRT implementation for the calculation of branch supports as Chi-squared based support. Species name is followed by protein accession number and by a two letter description of the larger taxonomic group (BA—Basidiomycota, Agaricomycotina; BU—Basidiomycota, Ustilaginomycotina; BP—Basidiomycota, Pucciniomycotina; BW—Basidiomycota, Wallemiomycotina; AS—Ascomycota, Saccharomycotina; AP—Ascomycota, Pezizomycotina). Green—Basidiomycota, red—Saccharomycotina, blue—Pezizomycotina.

The phylogeny of NRAMP transporters (Figure 6) did not indicate any duplication events predating the separation of asco- and basidiomycetes, but detected several more recent duplications, particularly in species of Saccharomycotina (where three duplications led to genes SMF1-3 in *S. cerevisiae*) and *Puccinia graminis*. Interestingly, only one NRAMP transporter gene was found in *H. werneckii*, although this species contains nearly every other gene in two copies due to its diploid genome formed by hybridization [39].

## 4. Discussion

Some fungal species can tolerate extremely high concentrations of salt as successfully as halophilic prokaryotes, and for certain salts, such as MgCl_2_, they even surpass them [15]. Although substantial progress has been made in studies of halophilic fungi, including recent whole genome sequencing projects, the genetic basis of fungal salt tolerance is only partially understood. An important reason for this is the complexity of the halotolerant phenotype and its interrelatedness with responses to other types of stress. For example, high environmental salinity triggers oxidative stress, while, in turn oxidative stress triggers the high osmolarity glycerol signaling pathway, which is crucial also for response to hypersaline conditions [40,41]. In the halotolerant yeast *Debaryomyces hansenii* (but not in *S. cerevisiae*) NaCl was shown to exert a protective effect against externally triggered oxidative stress [42]. In extremely halotolerant *H. werneckii* the expression of genes involved in oxidative damage protection increases at high salinity [18], where oxidative stress may be one of the growth limiting factors [16].

In order to investigate a possible link between the presence and redundancy of genes involved in oxidative stress response and halotolerance in fungi, we identified these genes in the genomes of three halotolerant/halophilic fungal species and compared them with the genomes of 16 other asco- and basidiomycetous fungi with varying degrees of halotolerance. Our results show that all species included in the analysis contained at least one peroxiredoxin, while both Ustilaginomycotina species lacked a catalase and *W. ichthyophaga* lacked a bifunctional catalase-peroxidase (Figure 1). The latter enzyme was present in only nine (of 19) species and is thought to be descended from a horizontal gene transfer event between Bacteroidetes and an ancestor of the ascomycetes [43]—although its identification also in the basidiomycete *Ustilago maydis* suggests an earlier transfer event.

Phylogenetic analyses of superoxide dismutases, catalases, and peroxiredoxins (Figure 2, Figure 3 and Figure 4) and also of thioredoxins and glutathione transferases (Supplementary Materials) showed a complex evolutionary history of these enzymes with several ancient and much more recent duplications (and gene losses). Most of the resulting phylogenetic clusters could be identified based on the *S. cerevisiae* proteins in each cluster. In several cases predicted proteins from *H. werneckii* and *A. pullulans* were relatively far from homologues of related fungi, such as proteins in a lineage of superoxide dismutases close to cytosolic copper-zinc superoxide dismutases (Figure 2), or in the case of two catalases from *H. werneckii* in an otherwise basidiomycetous phylogenetic cluster. Nevertheless, a BLAST search of these proteins against the GenBank database confirmed their putative function and the presence of amino acid domains characteristic for the given enzyme family. If this functional annotation is correct, *A. pullulans* and *H. werneckii* contain the highest cumulative number of proteins from the major three enzyme groups (superoxide dismutases, catalases, and peroxiredoxins) among all compared fungi (18 and 17 per haploid genome, respectively), while *M. globosa*, *U. maydis*, and *W. ichthyophaga* has the lowest number (4, 6, and 8, respectively).

The relatively low number of oxidative stress response genes in *W. ichthyophaga* is surprising in light of the interrelatedness of salt and oxidative stress. The species, however, is peculiar from many other aspects as well: (i) an isolated phylogenetic position (in a subphylum with only eight known species [13,14,44]); (ii) an absolute requirement for low water activity media and preference for NaCl compared to non-ionic osmolytes [44]; (iii) a compact genome only 9.6 Mbp large and with only 4884 predicted genes [21]; and (iv) transcriptional unresponsiveness and low expression of homologues of —in other fungi crucial— salt stress response genes such as transporters of salt ions [21].

It appears that the halophilic strategy of *W. ichthyophaga*, the most halophilic fungus known, is fundamentally different from the halotolerance observed in other fungal species. Consistent with this, the species is able to grow at salinities lethal to a majority of other organisms with only a moderate number of major enzymes involved in the response to oxidative stress. On the one hand *W. ichthyophaga* might employ other mechanisms for defense against ROS. Despite the genome reduction it retained the NRAMP manganese transporter (Figure 6), important for the maintenance of the intracellular antioxidant manganese pool [10]. On the other hand, the moderate number of antioxidant enzymes may be a consequence of low levels of oxidative stress in this fungus. While even the most osmotolerant fungi usually grow optimally in normal mycological media without added salt (with very few exceptions), the growth optimum of *W. ichthyophaga* is around 17% NaCl (w/v), indicating that the cells do not experience stress at this very high salinity. Even in these conditions the shortest observed generation time of the species is 15 hr [45]. The corresponding slow metabolism and the high salinity optimum could mean that the amount of ROS generated in *W. ichthyophaga* in hypersaline conditions is much lower than in other fungi exposed to the same environment. 

All other investigated species are able to grow in media without added salt. In these species, the numbers of superoxide dismutases, catalases and peroxiredoxins per haploid genome correlated with the maximum tolerated salinity (*p* < 0.05) (Figure 5). While the correlation was not strong, it supports the initial hypothesis about the link between salt and oxidative stress tolerance and warrants further research of the topic.

Further studies should attempt to overcome the limitations of this work, three of them in particular. Firstly, salinity limits of this study were recovered from various publications (and for some species these limits were not available); maximum tolerated salinity depends not only on the species in question, but also on the composition and form (liquid or solid) of the medium, growth temperature and other conditions, which means that halotolerance data from different sources are not necessarily comparable. Secondly, the number of gene copies encoding a certain antioxidant enzyme are only a partial indication of the oxidative stress tolerance, which does not take into account the expression and activity of the enzyme. These could be evaluated for example by direct measurements of the antioxidant capacity of cells or cell extracts. Thirdly, oxidative stress is not relevant only in hypersaline conditions, but also in many other environments and scenarios, thereby making the identification of a simple “antioxidant capacity-halotolerance” relationship much more difficult. A good antioxidant response is important for plant pathogens and of the here investigated species antioxidant enzymes have been linked to pathogenicity for example in *Magnaporthe oryzae* [46] and *U. maydis* [47]. From this point of view a plant pathogen, even if very salt-sensitive, is not ideal for comparison. As more well-annotated genomes become available, more optimal selection of species for comparative genomics of halotolerant fungi will be possible.

Finally, non-enzymatic antioxidant defenses should be considered as a possible alternative mechanism to enzymes for combating salt-induced oxidative stress. Intracellular manganese forming antioxidant complexes with small metabolites was shown to predict the radiation resistance of cells [9] and these metabolites were found to be much more important for combating radiation induced oxidative stress than enzymes such as superoxide dismutases [6,8,9]. Since non-enzymatic mechanisms are difficult to explore with genomic tools, we analyzed the homologues of transmembrane manganese transporters of the NRAMP family (Figure 6) as a possible indirect indicator of the cellular antioxidant manganese pool [10]. In the analysis presented here, no correlation between the number of NRAMP transporters and halotolerance was observed (Figure 5). However, the predictive power of the number of NRAMP transporter genes may be very low, as gene duplication is possibly limited by the cytotoxicity of intracellular Mn(III) accumulation [48].

In conclusion, the presented results support the suggestion that the most halophilic fungus known, *W. ichthyophaga*, employs a specialized strategy of survival at extreme concentrations of salt not seen in other fungi and can survive salinities detrimental to most other organisms with a moderate number of oxidative stress response genes. In other investigated fungal species, the maximum tolerated salinity correlated with the number of genes for superoxide dismutases, catalases and peroxiredoxins, three major enzymes of the cellular oxidative stress defense. This observation warrants further research into the link between the (enzymatic and non-enzymatic) antioxidant capacity of cells and their halotolerance.

## Figures and Tables

**Table 1:** Fungal species analyzed in this study and their maximum tolerated NaCl concentrations used in the correlation analyses.

Species	Phylogenetic Position	Maximum Tolerated Concentration of NaCl (w/v)
*Saccharomyces cerevisiae*	Ascomycota, Saccharomycotina	10 (our unpublished data)
*Debaryomyces hansenii*	Ascomycota, Saccharomycotina	24 [26]
*Candida albicans*	Ascomycota, Saccharomycotina	12.2 [27]
*Neurospora crassa*	Ascomycota, Pezizomycotina	12.8 [28]
*Magnaporthe oryzae*	Ascomycota, Pezizomycotina	8 [29]
*Sclerotinia sclerotiorum*	Ascomycota, Pezizomycotina	N.D. ^1^
*Aspergillus flavus*	Ascomycota, Pezizomycotina	10 [30]
*Penicillium rubens*	Ascomycota, Pezizomycotina	N.D. ^1^ (20 for related *P. notatum* and *P. chrysogenum* [30])
*Pyrenophora teres*	Ascomycota, Pezizomycotina	N.D. ^1^
*Parastagonospora nodorum*	Ascomycota, Pezizomycotina	N.D. ^1^
*Cryptococcus neoformans var. neoformans*	Basidiomycota, Agaricomycotina	16 (our unpublished data)
*Laccaria bicolor*	Basidiomycota, Agaricomycotina	N.D. ^1^
*Schizophyllum commune*	Basidiomycota, Agaricomycotina	7 [31]
*Malassezia globosa*	Basidiomycota, Ustilaginomycotina	N.D. ^1^ (5.5 for related *M. furfur* [32])
*Ustilago maydis*	Basidiomycota, Ustilaginomycotina	3.5 [33]
*Puccinia graminis*	Basidiomycota, Pucciniomycotina	N.D. ^1^
*Hortaea werneckii*	Ascomycota, Pezizomycotina	28 [12]
*Aureobasidium pullulans*	Ascomycota, Pezizomycotina	17 [16]
*Wallemia ichthyophaga*	Basidiomycota, Wallemiomycotina	35.9 [12]

^1^ N.D.—no data was found in the literature.

## Supplementary Materials

The following are available online at http://www.mdpi.com/2073-4425/9/3/143/s1. Supplementary File: Phylogenetic trees of genes encoding enzymes involved in oxidative stress response.
