# Supplementary material for: Overview of Oxidative Stress Response Genes in Selected Halophilic Fungi
Source: Genes (Basel). 2018 Mar 6;9(3):143. doi: 10.3390/genes9030143 (PMC5867864; doi:10.3390/genes9030143)

Supplementary File to the article:
Overview of oxidative stress response genes in selected halophilic fungi
by
Cene Gostinčar and Nina Gunde-Cimerman

Maximum likelihood phylogenies of genes involved in oxidative stress response. The phylogeny was estimated by PhyML software with aLRT implementation for the calculation of branch supports as Chi2 based support. Species name is followed by protein accession number and by a two letter description of the larger taxonomic group (BA - Basidiomycota, Agaricomycotina; BU - Basidiomycota, Ustilaginomycotina; BP - Basidiomycota, Pucciniomycotina; BW - Basidiomycota, Wallemiomycotina; AS - Ascomycota, Saccharomycotina; AP - Ascomycota, Pezizomycotina). Green - Basidiomycota, red - Saccharomycotina, blue - Pezizomycotina.

Glutathione peroxidases

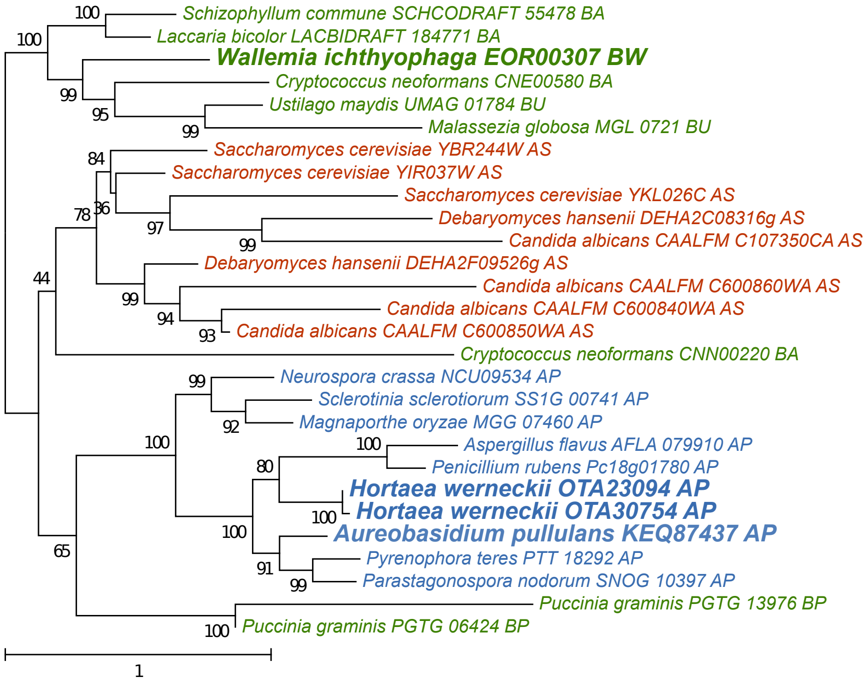

Glyoxalases I

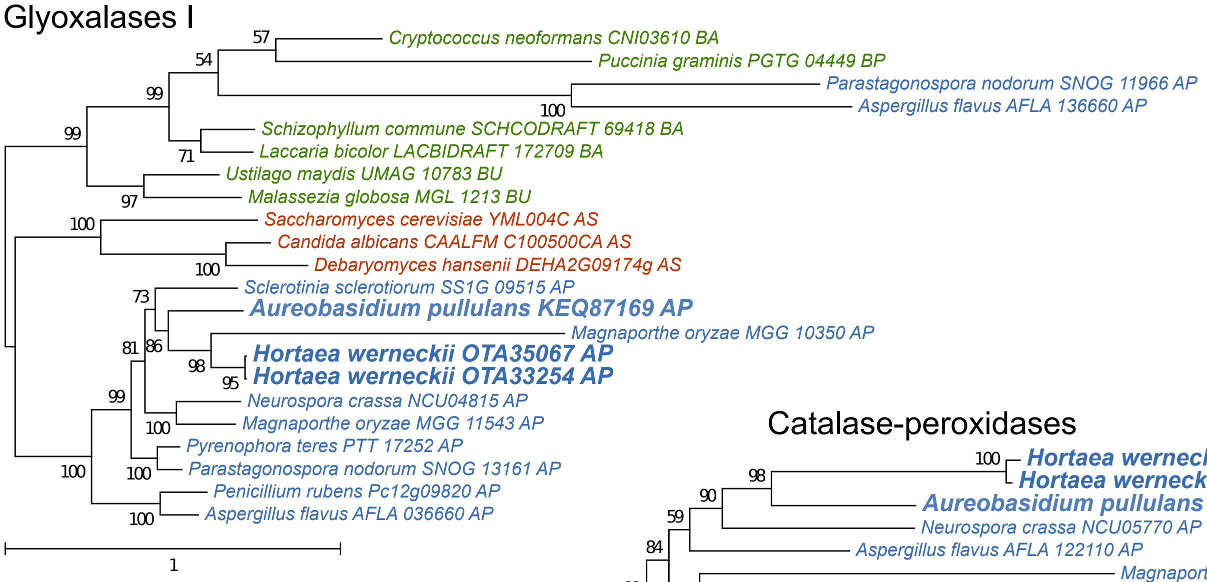

Catalase-peroxidases

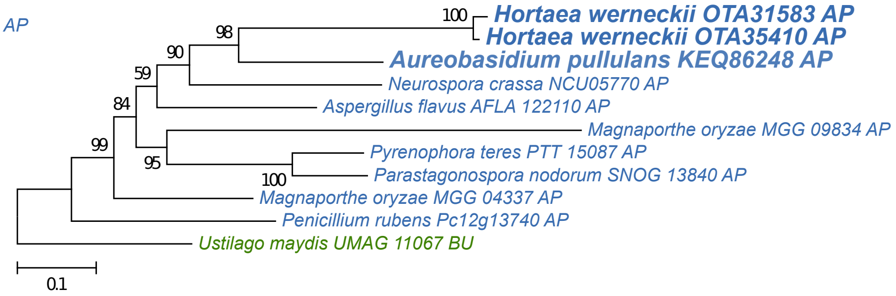

Glyoxalases II

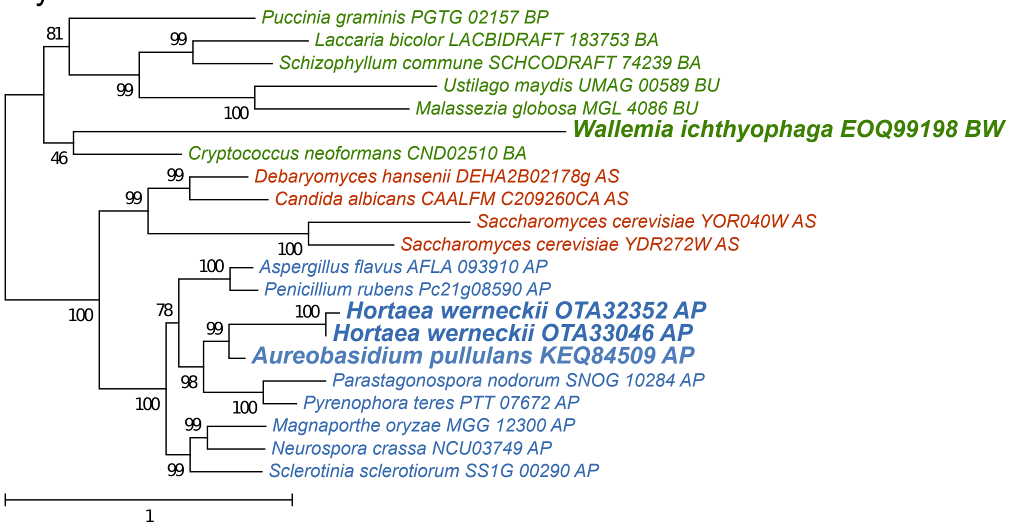

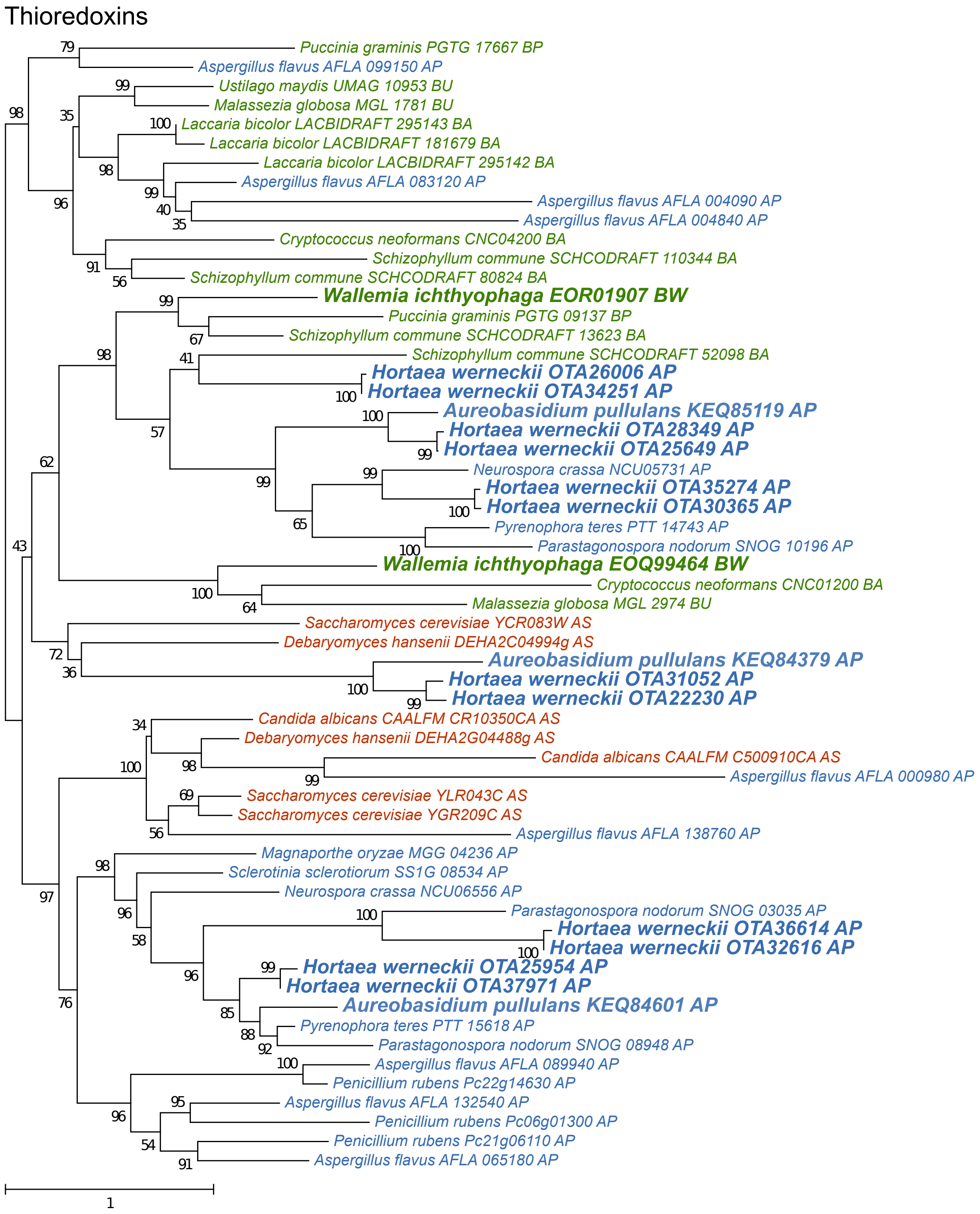
Thioredoxins
Puccinia graminis PGTG 17667 BP
Aspergillus flavus AFLA 099150 AP
Ustilago maydis UMAG 10953 BU
Malassezia globosa MGL 1781 BU
Laccaria bicolor LACBIDRAFT 295143 BA
Laccaria bicolor LACBIDRAFT 181679 BA
Laccaria bicolor LACBIDRAFT 295142 BA
Aspergillus flavus AFLA 083120 AP
Aspergillus flavus AFLA 004090 AP
Aspergillus flavus AFLA 004840 AP
Cryptococcus neoformans CNC04200 BA
Schizophyllum commune SCHCODRAFT 110344 BA
Schizophyllum commune SCHCODRAFT 80824 BA
Wallemia ichthyophaga EOR01907 BW
Puccinia graminis PGTG 09137 BP
Schizophyllum commune SCHCODRAFT 13623 BA
Schizophyllum commune SCHCODRAFT 52098 BA
Hortaea werneckii OTA26006 AP
Hortaea werneckii OTA34251 AP
Aureobasidium pullulans KEQ85119 AP
Hortaea werneckii OTA28349 AP
Hortaea werneckii OTA25649 AP
Neurospora crassa NCU05731 AP
Hortaea werneckii OTA35274 AP
Hortaea werneckii OTA30365 AP
Pyrenophora teres PTT 14743 AP
Parastagonospora nodorum SNOG 10196 AP
Wallemia ichthyophaga EOQ99464 BW
Cryptococcus neoformans CNC01200 BA
Malassezia globosa MGL 2974 BU
Saccharomyces cerevisiae YCR083W AS
Debaryomyces hansenii DEHA2C04994g AS
Aureobasidium pullulans KEQ84379 AP
Hortaea werneckii OTA31052 AP
Hortaea werneckii OTA22230 AP
Candida albicans CAALFM CR10350CA AS
Debaryomyces hansenii DEHA2G04488g AS
Candida albicans CAALFM C500910CA AS
Aspergillus flavus AFLA 000980 AP
Saccharomyces cerevisiae YLR043C AS
Saccharomyces cerevisiae YGR209C AS
Aspergillus flavus AFLA 138760 AP
Magnaporthe oryzae MGG 04236 AP
Sclerotinia sclerotiorum SS1G 08534 AP
Neurospora crassa NCU06556 AP
Parastagonospora nodorum SNOG 03035 AP
Hortaea werneckii OTA36614 AP
Hortaea werneckii OTA32616 AP
Hortaea werneckii OTA25954 AP
Hortaea werneckii OTA37971 AP
Aureobasidium pullulans KEQ84601 AP
Pyrenophora teres PTT 15618 AP
Parastagonospora nodorum SNOG 08948 AP
Aspergillus flavus AFLA 089940 AP
Penicillium rubens Pc22g14630 AP
Aspergillus flavus AFLA 132540 AP
Penicillium rubens Pc06g01300 AP
Penicillium rubens Pc21g06110 AP
Aspergillus flavus AFLA 065180 AP
1

Glutathione transferases

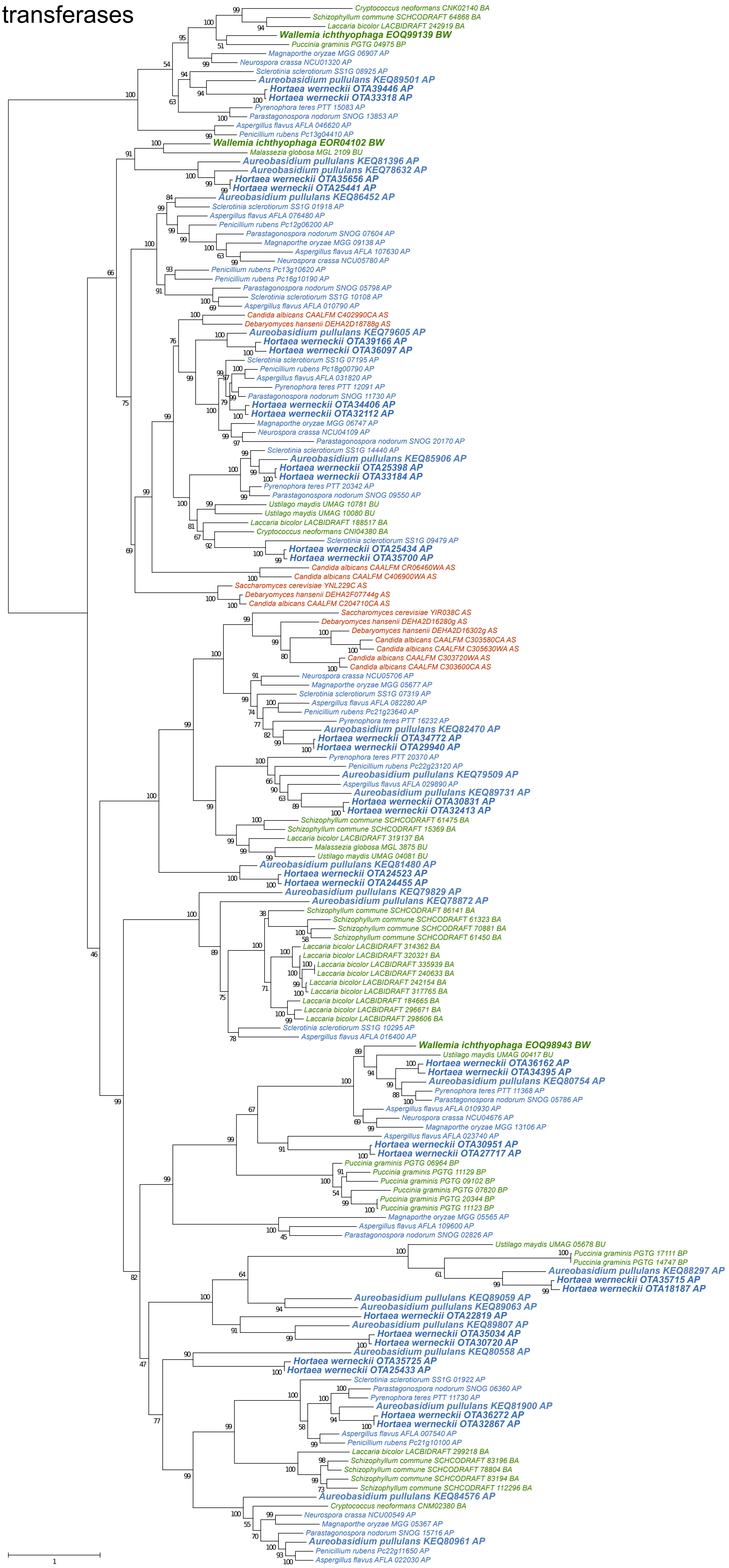

Supplement: Supplementary file 1 [file genes-09-00143-s001.pdf]
